# Evolution of microstructure and formation mechanism of Nd-Fe-B nanoparticles prepared by low energy consumption chemical method

**DOI:** 10.1039/c8ra08271e

**Published:** 2018-11-19

**Authors:** Yaozu Guo, Dong Zhao, Junhua You, Wenli Pei, Yingdong Qu, Xiaoyang Wang, Qingyu Meng

**Affiliations:** School of Materials Science and Engineering, Shenyang University of Technology Shenyang 110870 P. R. China youjunhua168@163.com; Key Laboratory for Anisotropy and Texture of Materials, Ministry of Education, Northeastern University Shenyang 110819 P. R. China peiwl@atm.neu.edu.cn

## Abstract

Nd_2_Fe_14_B nanoparticles were successfully prepared by using a low-energy chemical method. The microscopic characteristics and formation mechanisms of the phases were investigated at each stage during the preparation of Nd-Fe-B nanoparticles. The Nd-Fe-B intermediates, Nd-Fe-B oxides and reduced Nd-Fe-B nanoparticles were detected and analyzed by using TEM, STEM, XRD, SEM, VSM and Rietveld calculations. The results showed that the intermediate of Nd-Fe-B consisted of Fe_3_O_4_ and Nd and Fe elements surrounded by nitrile organic compounds. The Nd-Fe-B oxide was composed of NdFeO_3_ (48.619 wt%), NdBO_3_ (31.480 wt%) and α-Fe (19.901 wt%), which was formed by the reaction among Nd, Fe_3_O_4_ and B_2_O_3_. NdFeO_3_ and NdBO_3_ exhibited a perovskite-like lamellar structure, and the grain size was smaller than that of α-Fe. Nd-Fe-B particles were mainly composed of Nd_2_Fe_14_B and α-Fe phases. The small particles of NdFeO_3_ and NdBO_3_ and the interstitial position between oxide particles and α-Fe were more favorable for the formation of Nd_2_Fe_14_B particles. At the same time, the surface of α-Fe particles can also diffuse to form Nd_2_Fe_14_B nanoparticles. The coercivity of Nd-Fe-B particles was 5.79 kOe and the saturation magnetization was 63.135 emu g^−1^.

## Abstract 2

The Nd-Fe-B intermediate was successfully prepared by using a low-energy chemical method.

## Graphical abstract

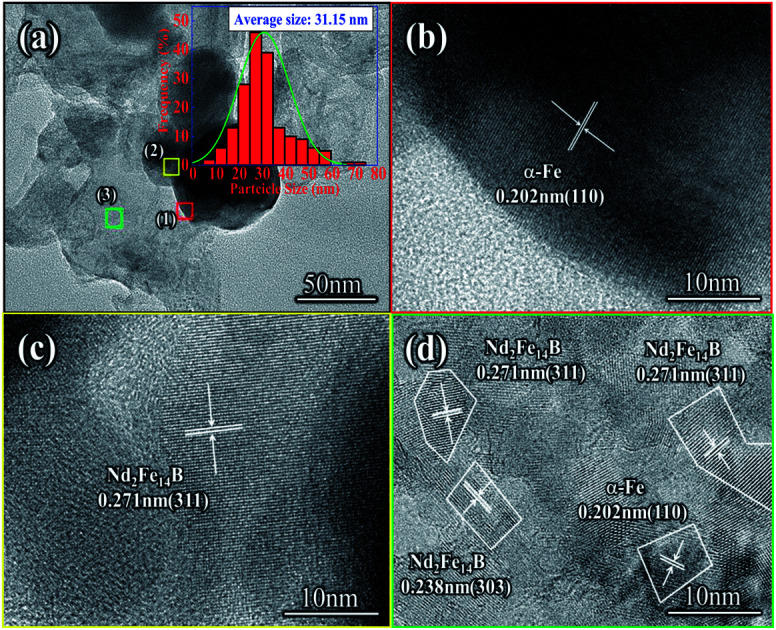

## 1. Introduction

In recent years, synthesis of magnetic nanostructures has been investigated for various permanent magnet (PM) applications due to their unique structural, electrical and magnetic properties.^1–4^ PMs are widely used in modern energy conversion devices, such as electric vehicles, wind turbine generators, information storage, energy conversion systems, *etc.*^5–10^ In particular, the Nd-Fe-B based PMs present the best magnetic properties, which can make these devices and systems smaller, lighter and more energy efficient.^11,12^ At present, the main methods for preparing Nd-Fe-B alloys are metallurgical methods,^13^ such as arc melting,^14,15^ high energy ball milling,^16^ mechanical alloying^17^ and rapid quenching.^18^ However, there are many disadvantages with these preparation methods. For example, the process is complex, the production cost is expensive, and the particle size distribution of the prepared powder is wide and irregular.

To avoid these shortcomings, scientists have investigated top-down synthetic methods, such as chemical synthesis.^19^ The chemical synthesis method has many advantages, including simple process, easy operation, and low cost. Moreover, the synthesis method can control the size of Nd-Fe-B nanoparticles by regulating the size of Nd-Fe-B intermediate grains.^20,21^ P. K. Deheri *et al.*^22^ reported a preparation method for Nd_2_Fe_14_B in which the precursor of Nd-Fe-B was prepared by sol–gel method, then burned to form Nd-Fe-B oxide and finally reduced to Nd_2_Fe_14_B nanoparticles by CaH_2_. From the point of view of reaction thermodynamics, some chemical reactions were proposed, and finally Nd_2_Fe_14_B/α-Fe exchange coupled nanoparticles were prepared. The coercivity of the product was as high as 3.9 kOe. Subsequently, P. K. Deheri *et al.*^23^ continued this research using the sol–gel method, and further proposed that there were two kinds of competitive reaction mechanisms in this method, and then improved the efficiency of the combined reaction and purity of Nd_2_Fe_14_B by adding amorphous B. Finally, the magnetic properties were increased to 9.125 kOe. H. X. Ma *et al.*^24^ proposed a nitrate based method for the preparation of Nd_2_Fe_14_B nanoparticles. In this method, Nd-Fe-B oxides were prepared by using the combustible characteristics of nitrate itself, and Nd_2_Fe_14_B nanoparticles were successfully prepared by CaH_2_ reduction. The coercivity of the product was 3.283 kOe. On the basis of the above methods, V. Swaminathan *et al.*^25^ improved the method and prepared Nd-Fe-B oxide and Nd-Fe-B nanoparticles by microwave assisted combustion. Based on the works of the above two teams, H. Parmar *et al.*^26^ changed the microwave power and further improved the technology. Consequently, Nd_2_Fe_14_B nanoparticles were prepared with coercivity of 8 kOe.

Low energy consumption chemical method is a potential preparation method for Nd-Fe-B nanoparticles. The greatest advantage of this method is that the chemical preparation process is simpler than the above methods. J. H. Jung^27^ first reported the feasibility of this method, but further developments on this method have not been reported since then. Moreover, some details of this method are still not clear, such as the reaction mechanism and evolution process of microstructure. In this paper, Nd-Fe-B nanoparticles were prepared by an optimized low energy chemical method. The following issues were investigated in depth: (1) the formation mechanism of the Nd-Fe-B intermediate; (2) the change in microstructure after annealing and reduction diffusion; (3) the structures of Nd-Fe-B oxide and Nd_2_Fe_14_B nanoparticles. This work is expected to provide better understanding of the preparation of Nd_2_Fe_14_B nanoparticles by low energy consumption chemical method.

## 2. Experimental procedure

### 2.1 Synthesis of Nd-Fe-B intermediates by chemical method

Nd-Fe-B nanoparticles were prepared by low energy consumption chemical method. In a typical procedure, 60 mL of oil ammonia (Aladdin, 80–90%) solution was added to a three-necked flask. Then, 4.5 mmol of neodymium acetylacetonate (Nd(acac)_3_, ≥98%) and 4.5 mmol of iron acetylacetonate (Fe(acac)_3_, ≥99%) were dissolved in oil ammonia in a glove box filled with high-purity argon. The solution was heated at 50 °C for 20 min, and then heated to 120 °C for 60 min to remove physically adsorbed water. Then, 2.5 mmol (C_2_H_5_)_3_NBH_3_ (97%, 0.5 g mL^−1^) was injected into the solution quickly and the condenser tube was attached to the flask. After this, the temperature was raised to 350 °C. The reaction mixture was stirred continuously for 90 min, and then cooled down to room temperature. The Nd-Fe-B intermediate was obtained as black particles after centrifugation with ethanol/*n*-hexane mixed solution and stored in *n*-hexane solution until further analyses.

### 2.2 Preparation of Nd-Fe-B oxides by annealing

The Nd-Fe-B intermediate was placed in an alumina crucible and transferred to a vacuum tube furnace under constant flow of 95% Ar/5% H_2_ gas. The particles were heated at 120 °C for 60 min to remove the residual *n*-hexane and ethanol. Then, the furnace was heated to 750 °C at 5 °C min^−1^ and held at this temperature for 120 min, after which the furnace was cooled naturally to room temperature. This process resulted in the formation of grey and black particles of Nd-Fe-B oxides.

### 2.3 Preparation of Nd_2_Fe_14_B nanoparticles by reductive diffusion of Nd-Fe-B oxides

Nd-Fe-B oxide particles were first ground in a glove box for 20 min. Then, CaH_2_ (Aladdin, ≥95%) was added to the mixture at 1 : 1.5 wt% ratio and the whole mixture was additionally ground for another 10 min. Then, the powder was pressed into a compact pellet under the pressure of 160 MPa for 2 min. The pressed pellets were placed in alumina crucibles and transferred into a vacuum tube furnace. Then, the vacuum tube furnace was evacuated and flushed with high purity Ar gas (≥99.999%) three times. The temperature was increased to 930 °C for 120 min at the rate of 5 °C min^−1^, and then cooled to room temperature. The pellets were quickly transferred into the glove box, crushed, and then ground for 30 min. The ground sample was then washed with 200 mL deionized water to remove the by-product of CaO. Finally, the sample was rinsed with acetone to remove water and stored in *n*-hexane solution containing 20 μL of oil ammonia.

## 3. Characterization

The microstructure was observed by JEOL transmission electron microscope (JEOL TEM 2010, 200 kV) and X-ray energy dispersive spectrometer (EDS). Phase analysis was performed by X-ray diffraction (XRD) using Bruker (D8-Advance) diffractometer under Cu Kα radiation (*λ* = 0.154 nm) over the 2*θ* range of 20–80° with 0.01° step size and 5° min^−1^ scanning speed. The morphology and elemental composition of Nd-Fe-B oxide and Nd-Fe-B particles were analyzed by scanning electron microscope (SEM, Gemini SEM 300) coupled with EDS (operating at 15–20 kV). The XRD parameters of Nd-Fe-B oxides and Nd-Fe-B particles were refined by GASA software, and the content of each phase was determined by Rietveld method. The Nd_2_Fe_14_B cell structure was constructed by Diamond software. Room temperature magnetic measurements were performed up to 22 kOe using vibrating sample magnetometer (MicroSense VSM EZ9).

## 4. Results and discussion

### 4.1 Phase composition and formation principle of Nd-Fe-B intermediate

**Fig. 1:**
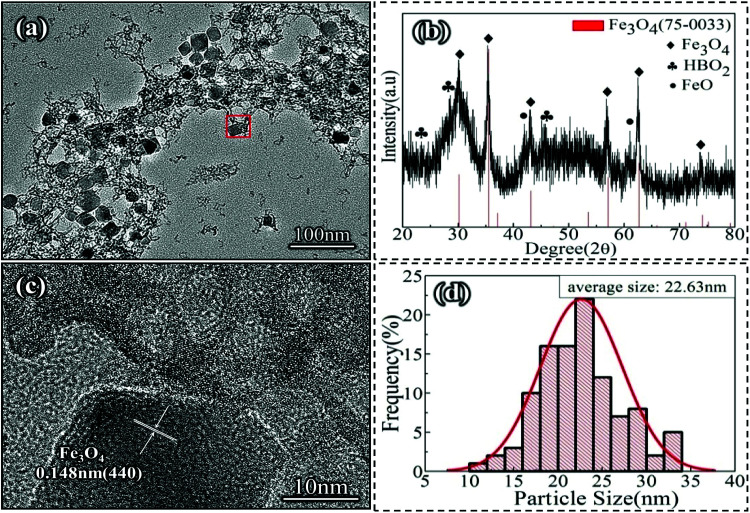
TEM image (a) and XRD pattern (b) of the Nd-Fe-B intermediate. (c): HRTEM image of red selected area in (a). (d): Particle size distribution of Nd-Fe-B intermediate.

It can be seen from Fig. 1(a) that the Nd-Fe-B intermediate mainly contains rhombohedral and cubic shaped large particles as well as flocculent substances. From the results of XRD analysis, Fe_3_O_4_ (75-0033) was found to be the main substance with higher crystallinity in Nd-Fe-B intermediate. In addition, there were small characteristic peaks of HBO_2_ (09-0015) and FeO (06-0615). The other substances were amorphous. HBO_2_, FeO and Fe_3_O_4_ were obtained from the thermal decomposition of (C_2_H_5_)_3_NBH_3_ and Fe(acac)_3_.^28^ The main reason for the formation of crystal particles in Fe_3_O_4_ was that Fe(acac)_3_ began to undergo thermal decomposition rapidly at 300 °C. Fe_3_O_4_ formed a cube or rhomboid depending on the heating rate of the intermediate. This is because the cubic phase and rhombic phase are formed in favor of Fe_3_O_4_ grains at the heating rate of 15–35 °C min^−1^ (ref. 29 and 30) when the typical Fe(acac)_3_ thermal decomposition occurs. The size distribution of the Fe_3_O_4_ grains was about 10 nm–35 nm, and the average particle size was about 22.63 nm. However, no phase associated with Nd was observed in either the XRD diffraction pattern or the TEM image. Therefore, the flocculent organic matter in Nd-Fe-B intermediate was further analysed, as shown in Fig. 1(c). It can be seen from the figure that the flocculent matter around the large Fe_3_O_4_ (0.148 nm (440)) grains showed a network structure. Also, the presence of fine grains with stripes can be observed in this structure. This indicates that the flocculent matter contained crystal particles.

**Fig. 2:**
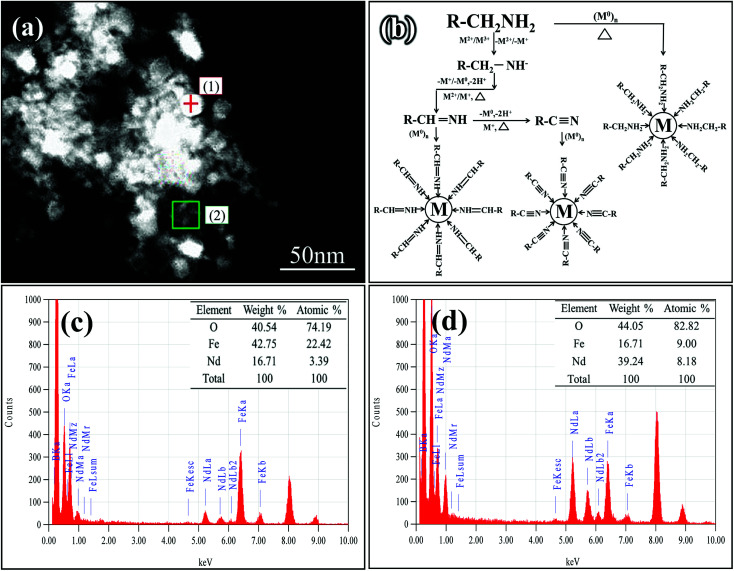
EDS analysis of the Nd-Fe-B intermediate phases. (a) SEM micrograph showing selected areas (1) and (2). (b): The schematic of the reactions. (c): Point-scan elements distribution and their percentages. (d): Surface scanned elements distribution and their percentages.

EDS analysis was employed to further analyze the elements present in organic compounds. The cubic region (1) and organic region (2) were selected for analysis in the Nd-Fe-B intermediate. The results are shown in Fig. 2(c) and (d). From the scan results, it was found that selected area (1) contained mainly three elements, Nd, Fe and O, with the atomic percentage of 3.39%, 22.42%, and 74.19%, respectively. It can be seen that in addition to O element, Fe was the main element in this area. Therefore, the cube was mainly Fe_3_O_4_ grain. Selected area (2) contained three main elements, Nd, Fe and O, with the atomic percentage of 8.18%, 9.00%, and 82.82%, respectively. It can be seen that the flocculent substances contained elements Nd and Fe in addition to organic matter. This is because, when the temperature reached 320 °C during the reaction process, (C_2_H_5_)_3_NBH_3_ also began to decompose in addition to acetylacetone.

Then, the (C_2_H_5_)_3_N–BH_3_ bond fractured and the H atom in BH_3_^−^ began to break apart (thermal decomposition temperature: 260–350 °C),^31–35^ which partly participated in the reduction of the oxide nucleus, to form the simple metal nucleus (M).^36,37^ Moreover, as the oleamine solution was weakly alkaline, the following reactions likely occurred:^38,39^

1 2BH_3_^−^ + 4H_2_O = 2BO_2_^−^ + 7H_2_↑

2 *n*BH_3_^−^ + 7M^*n*+^ + 7*n*OH^−^ = *n*BO_2_^−^ + 7M + 5*n*H_2_O

3 H_2_ + 2BO_2_^−^ = 2HBO_2_

Therefore, when Fe(acac)_3_ and Nd(acac)_3_ began to undergo thermal decomposition, some of the metal oxide particles were reduced to metallic elements by BH_3_^−^. Besides, oil ammonia may undergo metal ion-induced oxidation to nitriles, which can function as an entrapment agent to encapsulate metal elements, as reported by Chen and Osterloh's group.^40–45^ The mechanism of reaction involved in this experiment is shown in Fig. 2(b). Oil ammonia can be oxidized to nitriles by Nd and Fe particles, and encapsulated on the surface of metal, thus inhibiting the growth of Nd and Fe. Therefore, Nd and Fe elements can be detected in organic compounds. However, due to the small amount of (C_2_H_5_)_3_NBH_3_ and higher activity of Nd^3+^, BH_3_^−^ preferentially reacts with Nd^3+^. Consequently, some Fe_3_O_4_ grains could not be completely reduced, and larger Fe_3_O_4_ particles were formed as a result of further growth. In addition, a small amount of HBO_2_ was also formed (reaction (3)).

### 4.2 Characteristics and formation principle of Nd-Fe-B oxides

**Fig. 3:**
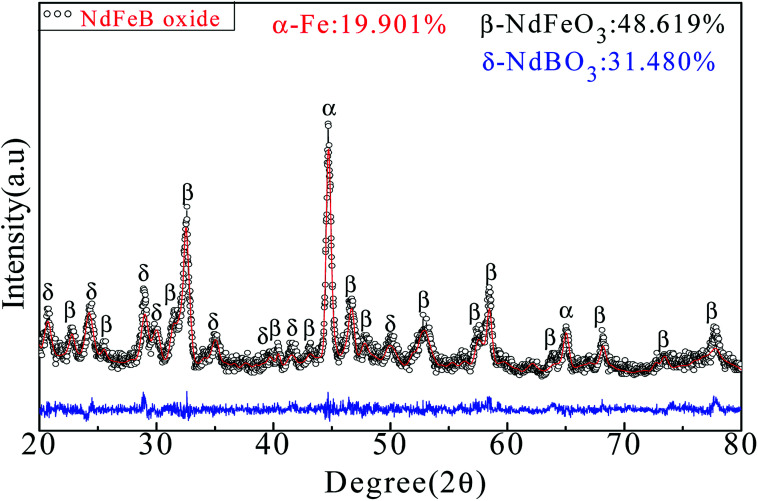
XRD pattern and Rietveld refinement of Nd-Fe-B oxides. Black points represent observed data and red line represents the fitted data. Blue line indicates the difference between calculated and observed data.

Heat treatment of the Nd-Fe-B intermediate at 750 °C under constant flow of 95% Ar/5% H_2_ gas resulted in the formation of oxide phases of NdFeO_3_, NdBO_3_ and α-Fe, as shown by the XRD pattern in Fig. 3. The results of Rietveld refinement indicate a mixture of three phases: 48.619 wt% of NdFeO_3_ (*a* = 0.5585 nm, *b* = 0.7758 nm, *c* = 0.5447 nm), 31.480 wt% of NdBO_3_ (*a* = 0.6298 nm, *b* = 0.6544 nm, *c* = 0.6546 nm), and 19.901 wt% of α-Fe (*a* = 0.2286 nm, *b* = 0.2286 nm, *c* = 0.2286 nm). The reactions involved in this process can be represented as follows:

4 Fe_3_O_4_ + 4H_2_ ≜ 3Fe + 4H_2_O

5 4Nd + 3Fe_3_O_4_ = 4NdFeO_3_ + 5Fe

6 2Nd + 3H_2_O + B_2_O_3_ = 2NdBO_3_ + 3H_2_↑

Due to the presence of a large number of Fe_3_O_4_ particles in the Nd-Fe-B intermediates, reaction (4) occurs at high temperature and Fe_3_O_4_ is reduced by H_2_ to form α-Fe. The organic matter decomposes with the removal of the flowing gas, and the elemental Nd is released, which reacts with Fe_3_O_4_ to form NdFeO_3_ at 750 °C. As the free energy (Δ*G*) of reaction (5) is −1888 kJ mol^−1^ (calculated by HSC Chemistry software), it is evident that reaction (5) can easily occur at 750 °C. At the same time, a small amount of HBO_2_ is dehydrated at high temperature to form B_2_O_3_. Then, B_2_O_3_ reacts with Nd to form NdBO_3_.^46^

**Fig. 4:**
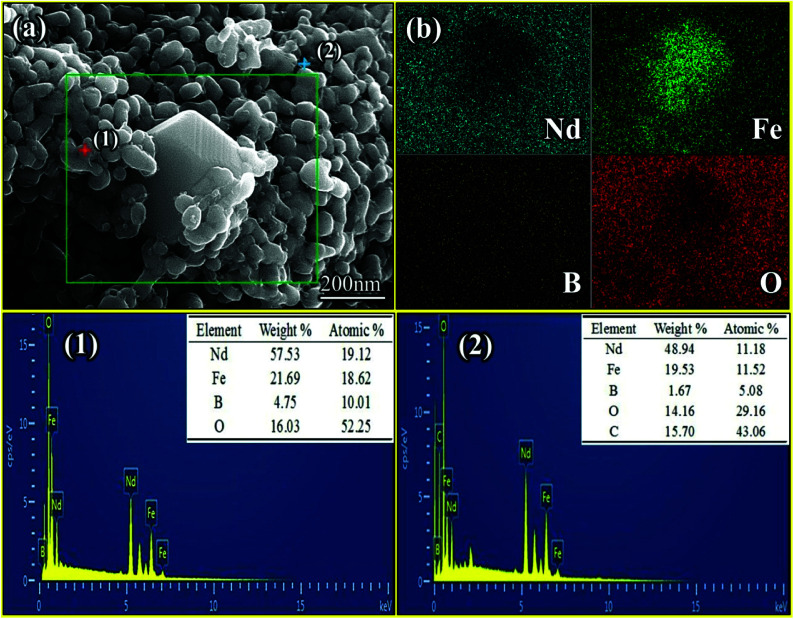
SEM images of the Nd-Fe-B oxides obtained from the Nd-Fe-B intermediates after annealing. (a) SEM image of the area selected for the EDS analysis shown in (b), (1) and (2) EDX analysis of selected areas (1) and (2).

Fig. 4(a) shows that Nd-Fe-B oxides were a mixture of large cubic particles and many lamellar particles. A large amount of flocculent material was also observed between grains. The elements of the selected region in Fig. 4(a) were further analyzed to distinguish the grains in the mixture. The results show that Fe element was the main element in the cube. Thus, the selected area was α-Fe particles, as shown in Fig. 4(b). The lamellar structure in selected area (1) was mainly composed of Nd, Fe, O and B (19.12, 18.62, 52.25, and 10.01 at%, respectively). According to Fig. 3, this region was mainly composed of NdFeO_3_ and NdBO_3_. In addition, NdFeO_3_ and NdBO_3_ showed lamellar structure of perovskite and pseudovaterite.^47,48^ These regions were mainly formed by reactions between tiny metallic grains generated after decomposition of organic matter. Thus, the NdBO_3_ and NdFeO_3_ grain sizes were relatively small after the reaction. The flocculent material of selected region (2) consisted mainly of elements Nd, Fe, O, C, and B (11.18, 11.52, 29.16, 43.06, and 5.08 at%, respectively), indicating that the region contained a lot of C-compounds. This is because organic matter was not completely decomposed at high temperatures to form carbides. Some carbides were also coated on the surface of the particles.

### 4.3 Characteristics of Nd-Fe-B powders obtained by reduction-diffusion

**Fig. 5:**
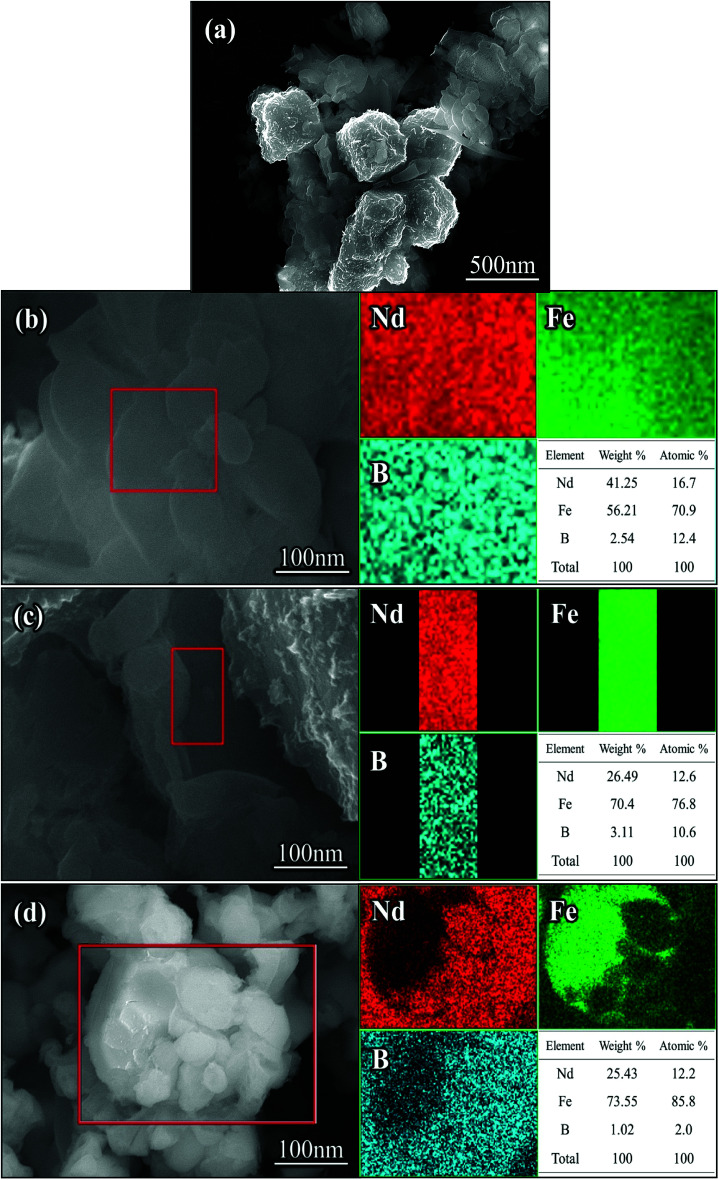
SEM images (a–d) and EDX-mapping of the Nd-Fe-B nanoparticles after removing CaO with deionized water.

After washing with deionized water, the reduced annealed powder mainly consisted of cubic particles and irregular small particles. In addition, there were some lamellar particles as shown in Fig. 5(a). The original dispersed particles formed blocks through the process of reduction and diffusion, and the lamellar structure grew further. The surface of the cube was wrapped in new phases, and the contiguous grain boundary began to fuse together at high temperatures.

EDS analysis was carried out in the regions of lamellar particles and cubic gaps. Inter-metallic uniform diffusion of Nd, Fe and B elements into each other was observed after the reduction treatment. Based on the atomic percentage, it was determined that the region was mainly Nd_2_Fe_14_B intermetallic compound, as shown in Fig. 5(b) and (c). It can be seen from the selected area in Fig. 5(d) that there were some irregular particles attached on the surface of the cubic grain. The Nd_2_Fe_14_B nanoparticles were formed on the surface and edges of the cubic α-Fe grains. After reduction, the Nd_2_Fe_14_B nanoparticles fell from the α-Fe grains, leaving behind pits on the surface and edges of the grains (see Fig. 5(d)). From EDS analysis of Fig. 5(d), it was evident that the cubic particle was α-Fe particle.

**Fig. 6:**
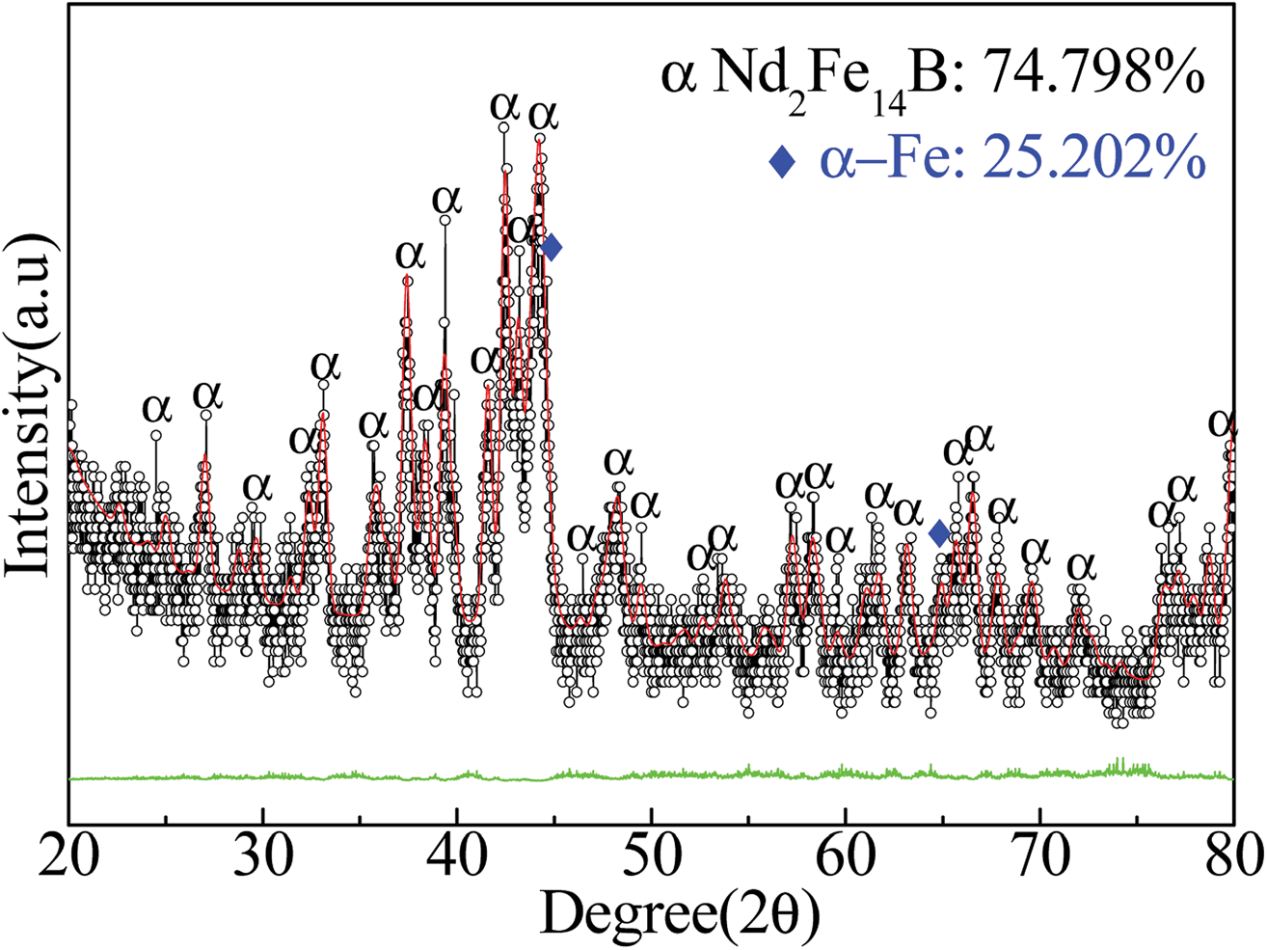
XRD pattern and Rietveld refinement result of Nd-Fe-B powders after reduction-diffusion at 930 °C, followed by removal of CaO byproduct.

Fig. 6 shows that the Nd-Fe-B powders after reduction-diffusion mainly contained two kinds of particles, 74.798 wt% of Nd_2_Fe_14_B (*a* = 0.8770 nm and *c* = 1.2160 nm) and 25.202 wt% of α-Fe (*a* = *b* = *c* = 0.290 nm). Nd_2_Fe_14_B lattice parameters calculated from Rietveld refinement were found to be *a* = 0.8770 nm and *c* = 1.2160 nm. These values were comparable with earlier reports.^21^ The following reactions likely occurred in the process.^48^

7 4NdFeO_3_ + 12CaH_2_ = 2H_5_Nd_2_ + 4Fe + 12CaO + 7H_2_↑

8 4NdBO_3_ + 12CaH_2_ = 2H_5_Nd_2_ + 4B + 12CaO + 7H_2_↑

9 2H_5_Nd_2_ + 28Fe + 2B = 2Nd_2_Fe_14_B + 5H_2_↑

Due to the strong reducibility of CaH_2_, NdFeO_3_ and NdBO_3_ were reduced to H_5_Nd_2_, Fe and B at high temperature. Reaction (7) occurred when the temperature was 310–360 °C, and reaction (8) occurred when the temperature reached 600 °C. Finally, there was a combination reaction (reaction (9)) between the phases to form Nd_2_Fe_14_B nanoparticles when the temperature reached 730 °C.^27,48^

**Fig. 7:**
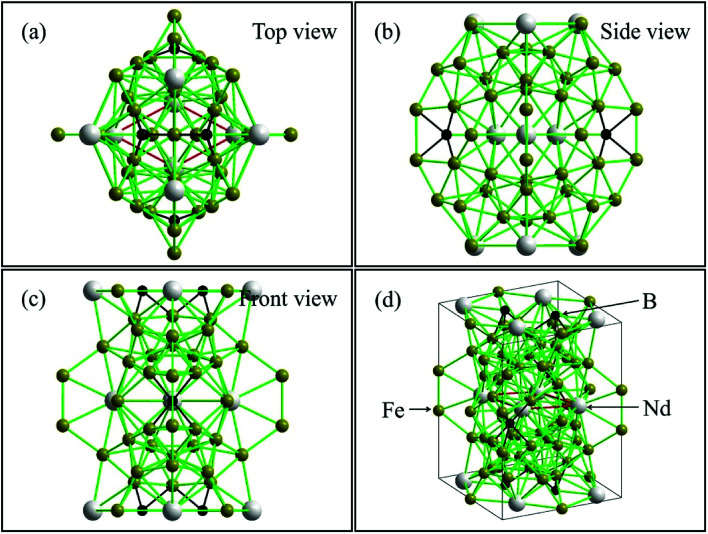
Structure of Nd_2_Fe_14_B by GASA, the values are obtained from Rietveld refined crystallographic parameters of Nd_2_Fe_14_B.

Fig. 7 shows that Nd_2_Fe_14_B cell was composed of four Nd_2_Fe_14_B units consisting of 68 atoms with a *P*4_2_/*mnm* structure. The Nd atoms were located in the three layers of unit cell, upper, middle and lower, with four atoms distributed per layer. The distance between the upper and lower Nd atoms was 5.373 Å. The nearest distance between Nd and Fe atoms was 3.111 Å and the distance between Fe and B atoms was 2.201 Å. The boron atoms were embedded in the octahedron center of the Nd and Fe atoms, and the B element was located in a plane at the center of the three Nd protons, as shown in Fig. 7(a). Due to the existence of magnetically active 3d elements (Fe) with the triggering presence of magnetic moments, the presence of the B atom greatly increased the possibility of mobile magnetism.^48^ The distance between the Nd atoms in the intermediate layer of the unit cell was 3.698 Å. The B atom was located at the center of the 8 iron atoms. The distance between Fe and B atoms was 2.08 Å, and the distance between Nd and B atoms was 2.794 Å. At this point, the B atom constructed a magnetic distance exchange bridge between the Nd and Fe atoms, which optimized the bonding ability between the atoms. This enhancement of the bonding ability helped to stabilize the structure.^49^ In addition, the distance between Fe and the nearest neighbor of Fe also affected the magnetic moment of Nd_2_Fe_14_B. The shortest distances between Fe and the surrounding Fe atoms in the cell structure were 2.382 Å and 2.443 Å. The shortest atomic distances in this study were slightly smaller than previously reported values (0.239 nm and 0.244 nm).^50^ This decrease in interatomic distance resulted in reduction of the intrinsic magnetic moment.

**Fig. 8:**
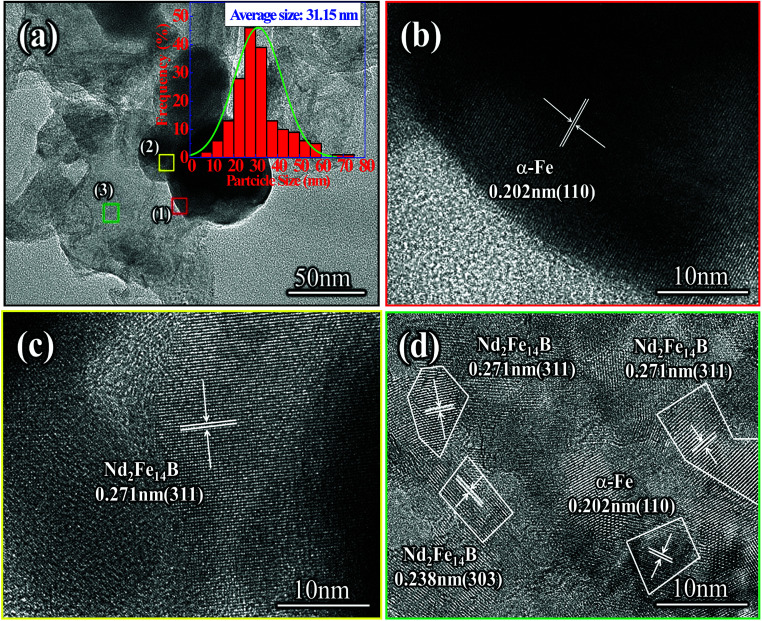
(a) TEM image of Nd_2_Fe_14_B/α-Fe nanoparticles produced by reduction-diffusion process, (b)–(d) HRTEM images of Nd_2_Fe_14_B/α-Fe nanoparticles.

As seen in Fig. 8(a), the size distribution range of the Nd-Fe-B nanoparticles with an average grain size of 31 nm was 5–75 nm. The size of the Nd-Fe-B nanoparticles was mainly concentrated in the range of 25–30 nm. Nd-Fe-B powder contained a small amount of large particles, and the surface of the particles was partially covered with defects. This was due to the formation of Nd_2_Fe_14_B nanoparticles on the surface and their subsequent removal, leaving behind the defects. The HRTEM image of the selected area (1) shows that the area contained elemental α-Fe particles with a crystal plane spacing of 0.202 nm, which can be assigned to the (110) lattice plane of α-Fe, Fig. 8(B). At the same time, HRTEM analysis of the interstitial region between the particles showed that the region was mainly composed of Nd_2_Fe_14_B, and the lattice spacing was 0.271 nm, which corresponded to the (311) plane of Nd_2_Fe_14_B, Fig. 8(c). The high resolution detection showed that the area (3) of powder block leveling had obvious polycrystalline properties. The crystal spacing between 0.271 nm and 0.238 nm was mainly the (311) and (303) planes of Nd_2_Fe_14_B, Fig. 8(d). The (110) lattice plane of α-Fe was also observed, and the crystal plane spacing was 0.202 nm. This is because the diffusion reaction occurred slowly and the powder composition after reduction was not uniformly distributed, which resulted in the diffusion reaction shifting towards the favorable direction. Therefore, a small amount of α-Fe nanoparticles were encapsulated in the center of the Nd_2_Fe_14_B block.

**Fig. 9:**
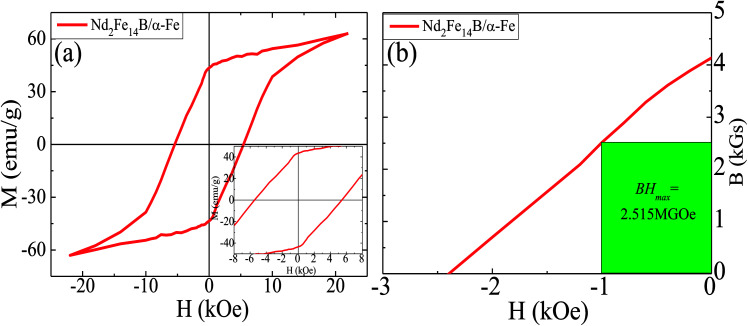
(a) Henkel plots of Nd_2_Fe_14_B/α-Fe synthesized sample. Inset: Enlarged view of magnetic hysteresis loops at low magnetic field. (b) Second quadrant *B*–*H* curves for the samples.

From the VSM results in Fig. 9, it can be seen that coercivity, saturation magnetization and the maximum magnetic energy product (*BH*)_max_ of Nd_2_Fe_14_B/α-Fe powder were 5.79 kOe, 63.135 emu g^−1^ and 2.515 MGOe, respectively. The remanence value was determined to be 45.071 emu g^−1^. As can be seen from the images in Fig. 8, there were also tiny regions of α-Fe located in the Nd_2_Fe_14_B block. Also, a small amount of α-Fe had a large grain size, which resulted in its incomplete reduction-diffusion reaction. At the same time, it can be seen from the demagnetization curve of the second phenomenon that the coercivity of the powder decreased rapidly when the applied magnetic field was reversed. This is because there were very few independent α-Fe particles in the powder. As the size of these soft magnetic particles was about 50 nm, their coupling effect with Nd_2_Fe_14_B hard magnetic particles was relatively weak. When the magnetic field was reversed, the magnetic moments of the α-Fe nanoparticles rotated rapidly, resulting in a rapid decrease in the coercivity of the sample. However, there was no obvious step in the hysteresis loop due to the presence of a large number of small size α-Fe nanoparticles. There are many small size nanocrystals in the sample, which is one of the reasons why the sample's coecivity is not so large. According to Feng *et al.*,^51^ when grain size is ≤20 nm, the coercivity noticeably decreases; Sun *et al.*^52^ further reported that when grain size ≤ 15 nm, the coercivity decreases rapidly, with a reasonable explanation given by theoretical calculation. In addition, some Nd-Fe-B nanoparticles were oxidized during the measurement of magnetic properties, which results into a lower (*BH*)_max_ in this sample.

## 5. Conclusions

In summary, Nd_2_Fe_14_B nanoparticles were successfully synthesized by a low-energy chemical method. The Nd-Fe-B intermediate was first synthesized and was composed of two parts, one of which was Fe_3_O_4_ nanoparticle, and the other part was composed of Nd and Fe elements encapsulated by nitrile organic matter. Nitrile organics were decomposed and Nd and Fe elements were released respectively during annealing. Moreover, Nd and Fe elements reacted with Fe_3_O_4_ and B_2_O_3_ nanoparticles to form NdFeO_3_ and NdBO_3_ phases with perovskite lamellar structure. In addition, some Fe_3_O_4_ grains were reduced to α-Fe phases. After reductive diffusion, there were a large number of lamellar Nd_2_Fe_14_B grains in the Nd-Fe-B nanoparticles because the NdBO_3_ and NdFeO_3_ small particles were reduced more easily during the process of reduction and diffusion. In addition, Nd_2_Fe_14_B nanoparticles were also formed on the surface of α-Fe grains by diffusion. The coercivity of Nd-Fe-B nanoparticles was as high as 5.79 kOe, and the saturation magnetization was 63.135 emu g^−1^.

## Conflicts of interest

The authors have no conflicts to declare.

## Supplementary Material
